# Many Putative Endocrine Disruptors Inhibit Prostaglandin Synthesis

**DOI:** 10.1289/ehp.1002635

**Published:** 2010-11-16

**Authors:** David M. Kristensen, Maria L. Skalkam, Karine Audouze, Laurianne Lesné, Christele Desdoits-Lethimonier, Hanne Frederiksen, Søren Brunak, Niels E. Skakkebæk, Bernard Jégou, Jacob B. Hansen, Steffen Junker, Henrik Leffers

**Affiliations:** 1 Department of Growth and Reproduction, Rigshospitalet, University of Copenhagen, Copenhagen, Denmark; 2 Center for Biological Sequence Analysis, Department of Systems Biology, Technical University of Denmark, Lyngby, Denmark; 3 Institut National de la Santé et de la Recherche Médicale (INSERM), Université de Rennes I, Campus de Beaulieu, Rennes, France; 4 Department of Biomedical Sciences, Panum Institute, University of Copenhagen, Copenhagen, Denmark; 5 Department of Human Genetics, University of Aarhus, Aarhus, Denmark

**Keywords:** antiandrogens, benzophenones, cyclooxygenase, endocrine disruptors, parabens, phthalates, PPARs, prostaglandins

## Abstract

**Background:**

Prostaglandins (PGs) play key roles in development and maintenance of homeostasis of the adult body. Despite these important roles, it remains unclear whether the PG pathway is a target for endocrine disruption. However, several known endocrine-disrupting compounds (EDCs) share a high degree of structural similarity with mild analgesics.

**Objectives and Methods:**

Using cell-based transfection and transduction experiments, mass spectrometry, and organotypic assays together with molecular modeling, we investigated whether inhibition of the PG pathway by known EDCs could be a novel point of endocrine disruption.

**Results:**

We found that many known EDCs inhibit the PG pathway in a mouse Sertoli cell line and in human primary mast cells. The EDCs also reduced PG synthesis in *ex vivo* rat testis, and this reduction was correlated with a reduced testosterone production. The inhibition of PG synthesis occurred without involvement of canonical PG receptors or the peroxisome proliferator–activated receptors (PPARs), which have previously been described as targets of EDCs. Instead, our results suggest that the compounds may bind directly into the active site of the cyclooxygenase (COX) enzymes, thereby obstructing the conversion of arachidonic acid to PG precursors without interfering with the expression of the COX enzymes. A common feature of the PG inhibitory EDCs is the presence of aromatic groups that may stabilize binding in the hydrophobic active site of the COX enzymes.

**Conclusion:**

Our findings suggest a hitherto unknown mode of action by EDCs through inhibition of the PG pathway and suggest new avenues to investigate effects of EDCs on reproductive and immunological disorders that have become increasingly common in recent decades.

Prostaglandins (PGs) belong to the group of short-lived lipid signaling compounds that are formed from arachidonic acid (AA), after its mobilization from membrane phospholipids by any of a broad array of stimuli (Smith et al. 2000). The molecules act locally in a paracrine or autocrine fashion and are involved in such processes as early male sexual development and masculinization, development of sexual behavior, induction of labor, inflammatory responses, pain, calcium movement, vasodilation, and hormone regulation (Adams and McLaren 2002; Amateau and McCarthy 2004; Gupta 1989; Gupta and Goldman 1986; Smith et al. 2000). Deregulation of the PG pathway has also been implicated in the pathophysiology of several diseases, such as cancer and cardiovascular and inflammatory diseases (FitzGerald 2003). In addition, prenatal inhibition of the pathway with acetaminophen/paracetamol (Ace), which belongs to the group of mild analgesics that are pharmaceutical cyclooxygenase (COX) enzyme inhibitors, has recently been associated with atopic diseases in childhood (Perzanowski et al. 2010; Rebordosa et al. 2008; Shaheen et al. 2002, 2005).

We recently noticed that phthalates, known endocrine-disrupting compounds (EDCs), share a high degree of structural similarity with salicylates such as aspirin [acetyl salicylate (ASA)] that inhibit the COX-mediated conversion of AA to PGs, and previous studies have indicated that certain phthalates could interfere with the pathway (Tavares and Vine 1985). In the present study, we investigated whether known EDCs could interfere with the PG pathway in a juvenile mouse Sertoli cell line, gestational day (GD) 14.5 fetal rat testes, and in primary human mast cells.

## Materials and Methods

### Cell culture and PG screen assay

SC5 mouse juvenile Sertoli cells (Hofmann et al. 1992) were cultured in Dulbecco’s modified Eagle’s medium with 10% fetal calf serum (FCS), 100 U/mL penicillin, 100 μg/mL streptomycin, and 1 mM l-glutamine (all from Invitrogen, San Diego, CA, USA) at 37ºC with 5% CO_2_. Only phthalate-free polystyrene flasks and 12-well plates (NUNC, Roskilde, Denmark) were used. The day before each experiment, 10^5^ SC5 cells were seeded in each well in a 12-well plate. The next day, media were removed and 1 mL fresh medium containing either a test compound or vehicle was added. Peroxisome proliferator–activated receptor-α (PPARα) and retinoid X receptor (RXR) agonists (613333 and LGD1069, respectively) were kindly provided by P. Sauerberg (Novo Nordisk, Bagsværd, Denmark); all other receptor ligands and EDCs (listed in Table 1) were purchased from Sigma-Aldrich (St. Louis, MO, USA) or Cayman Chemicals (Ann Arbor, MI, USA). The compounds were tested in at least three concentrations with the far left row in the plate containing the highest concentration and with decreasing concentrations toward the right side of the plate. Negative controls were always in the far right next to the lowest concentration of test compound. Twenty-four hours after exposure, media were removed and snap frozen on dry ice for later PG measurements, and cells were either harvested for RNA or used for cytotoxicity tests. To minimize the number of cells lost during medium changes, all pipetting was carried out with media added in droplets, and care was taken to minimize contact of the pipette tip with the bottom of the well.

### Culture and differentiation of primary human mast cells

Primary human mast cells were generated from CD133^+^ cells from buffy coat preparations as described previously (Holm et al. 2008). In brief, CD133^+^ cells were separated using the AC133 cell isolation kit and a magnetic LS^+^ separation column according to the manufacturer’s instructions (Miltenyi Biotech, Bergisch Gladbach, Germany). The purified CD133^+^ cells were suspended at 5 × 10^5^ cells/mL in StemSpan medium (Stem Cell Technologies, Vancouver, British Columbia, Canada) supplemented with 100 ng/mL human recombinant stem cell factor (rhSCF; R&D Systems, Abingdon, UK), 50 ng/mL human recombinant interleukin-6 (rhIL-6; R&D Systems), 1 ng/mL human recombinant interleukin-3 (rhIL-3; PeproTech, London, UK), and 100 μg/mL penicillin/streptomycin (GIBCO BRL, Grand Island, NY, USA) and grown for 3 weeks, after which rhIL-3 was omitted from the culture media. From week 6, 10% FCS (GIBCO BRL) was added, and mast cells were analyzed after 1 week. Cells were cultured in suspension for the entire period, and medium was renewed weekly.

Mature mast cells were sensitized by incubation with 2 μg/mL human myeloma IgE, kindly provided by L.K. Poulsen (National University Hospital, Copenhagen, Denmark) for 24 hr at 37ºC in StemSpan medium (Stem Cell Technologies) supplied with 100 ng/mL rhSCF and 50 ng/mL rhIL-6. The sensitized mast cells were washed, and 10^4^ cells were resuspended in 100 μL PIPES [piperazine-*N*,*N*′-bis(2-ethanesulfonic acid)] buffer with rhSCF and rhIL-6. Cells were activated by incubation with 100 μL anti-IgE (5 μg/mL; Dako, Glostrup, Denmark) for 30 min at 37ºC.

### Animals and culture of GD14.5 testes

Animal experiments were approved by the local ethics committee, and the animals were treated humanely with regard for alleviation of suffering. Pregnant female Sprague-Dawley rats bred in-house (Groupe d’Etude de la Reproduction Chez l’Homme et les Mammiferes–INSERM U625; Rennes, France) were anesthetized by intraperitoneal injection with 40 mg/kg sodium pentobarbital (Sanofi-Synthélabo, Libourne, France) on GD14.5. The testes were aseptically removed from male fetuses under a binocular microscope and then immediately explanted *in vitro*.

Testes were cultured on Millipore filters (0.45 μm pore size; Millipore Corp., Bedford, MA, USA), as previously described (Chauvigne et al. 2009; Habert et al. 1991; Lassurguere et al. 2003). Briefly, each GD14.5 fetal testis was removed with the adjacent mesonephros, placed on a filter floating in a culture dish on 0.5 mL M199 medium (Invitrogen) supplemented with 50 μg/mL gentamicin (Life Technologies, Cergy-Pontoise, France) and 2.5 μg/mL fungizone (Life Technologies), and incubated in a humidified atmosphere (5% CO_2_ at 37ºC) for 24, 48, or 72 hr. Two testes were cultured in 500 μL medium containing either vehicle [dimethyl sulfoxide (DMSO)] or a test compound. Half of the volume of the culture medium was refreshed every 24 hr.

### Electroporation

SC5 cells (8 × 10^5^) were electroporated using Amaxa Nucleofector (Lonza, Basel, Switzerland) in 100 μL electroporation buffer [20 mM HEPES, pH 7.0; 137 mM NaCl; 5 mM KCl; 0.7 mM Na_2_HPO_4_; 6 mM glucose; 0.1 mM β-mercaptoethanol) containing 10 μM or 100 μM mono-*n*-butyl phthalate (MBP), 10 μM di-*n*-butyl phthalate (DBP), or ethanol vehicle. Immediately after electroporation, cells were transferred to media without phthalate and cultured in 12-well plates. Cells were cultured 6 hr before media were harvested for prostaglandin D2 (PGD2) measurement.

### Cytotoxicity assay

After phthalate exposure, cells were counted and the cell number was compared with controls. We tested cytotoxicity of the phthalates after 24 hr exposure using a TOX-8 *In Vitro* Toxicology Assay Kit (Sigma Aldrich, St. Louis, MO, USA).

### Testosterone and PG measurement

Half the medium of each testis culture was recovered every 24 hr and stored at −80ºC until analysis by testosterone radioimmunoassay using a Coat-A-Count Total Testosterone Kit (Siemens, Los Angeles, CA, USA) without prior extraction. PGD2 and prostaglandin E2 (PGE2) were determined by Prostaglandin D2-MOX enzyme immunoassay (EIA) and Prostaglandin E2 EIA Kit–Monoclonal (Cayman Chemicals), respectively. The plates were read at 405 nM with a reference wavelength of 620 nM.

### Real-time polymerase chain reaction (PCR) analysis

We isolated RNA using the NucleoSpin RNA II purification kit with DNase I treatment as described by the manufacturer (Macherey-Nagel, Düren, Germany). One microgram of DNase I–treated RNA was reverse transcribed with avian myeloblastosis virus reverse transcriptase (USB Corp., Cleveland, OH, USA) using dT20 primers and random hexamers, and was ultimately resuspended in 100 μL Tris-EDTA buffer. Quantitative reverse transcriptase PCR (RT-PCR) analysis was performed in triplicate in a Stratagene Mx3000P system (Stratagene, La Jolla, CA, USA) with Brilliant SYBR Green QPCR Master Mix (Stratagene), using 35 cycles for amplification. PCR products were run on 2% agarose gels and visualized by ethidium bromide staining. Representative bands from each primer combination were excised and sequenced for verification (Eurofins MWG Operon, Ebersberg, Germany). Primers [see Supplemental Material, Table 1 (doi:10.1289/ehp.1002635)] were obtained from DNA Technology (Aarhus, Denmark).

### PPAR reporter and PPAR transactivation experiments and viral transduction

We performed PPAR response element reporter (TK-PPRE-luc) and PPAR transactivation (PPARδ-LBD/Gal4, PPARγ-LBD/Gal4, pM, and UAS-luc) experiments as described previously (Christensen et al. 2009; Hansen et al. 2001). SC5 cells (10^4^) were transfected using FuGENE HD (Roche, Basel, Switzerland) in 96-well plates with the plasmids and cytomegalovirus–*Renilla*. The experiments were initiated 24 hr after transfection, and cells were harvested 48 hr after transfection. Harvested cells were assayed for luciferase activity using Promega Dual-Luciferase Reporter Assay (Promega, Madison, WI, USA). Viral experiments were performed as described by Hansen et al. (2001) and Nielsen et al. (2008).

### Liquid chromatography–tandem mass spectrometry (LC-MS/MS)

All experiments were performed three times in triplicate in 12-well plates with 10^5^ cells in each well. After 24 hr, cells were exposed to 10 μM DBP, 10 μM MBP, 10 μM di-2-ethylhexyl phthalate (DEHP), 10 μM mono-2-ethylhexyl phthalate (MEHP), or vehicle. In addition, media containing phthalates were also incubated in 12-well plates without cells to test for possible contamination. The next day, cells were harvested by trypsinization, washed three times with phosphate-buffered saline, centrifuged to a pellet in an Eppendorf tube, and immediately frozen at −80ºC together with samples of the media. Methanol (60 μL) was added to each tube; pellets were then sonicated at 36ºC for 15 min and centrifuged. The supernatants were immediately transferred to phthalate-free glass tubes.

Monoester phthalates (MBP and MEHP) were measured as described by Frederiksen et al. (2008). The limits of detection (LODs) were 0.94 ng/mL and 0.18 ng/mL for MBP and MEHP, respectively. Secondary DEHP metabolites [mono-(2-ethyl-5-hydroxyhexyl) phthalate (5-OH-MEHP; LOD < 0.60 ng/mL), mono-(2-ethyl-5-oxohexyl) phthalate (5-OXO-MEHP; LOD < 0.14 ng/mL), and mono-(2-ethyl-5-carboxypentyl) phthalate (5-CX-MEPP; LOD < 0.43 ng/mL)] were also measured in SC5 cells, but levels were consistently < LODs.

### Molecular modeling of chemical binding to the COX active site of COX-2

We assessed molecular modeling on COX-2 protein by standard computer modeling studies using MOE 2007.09 (Chemical Computing Group Inc., Köln, Germany). We obtained the crystal structure of murine COX-2, which is very similar to human COX-2 (Kurumbail et al. 1996), from the Protein Data Bank (accession no. 1PXX; Research Collaboratory for Structural Bioinformatics 2010; Rowlinson et al. 2003). Each compound was docked using alpha triangle for the placement phase and London dG scoring for the scoring function.

### Statistical analysis

All results are presented as mean ± SE of all experimental replicates, except for quantitative RT-PCR, where results are presented as mean ± SD. We assessed statistical significance using a two-sided unpaired Student’s *t*-test; *p* < 0.05 indicates statistical significance.

## Results

### EDCs dose-dependently inhibit PG synthesis

**Figure 1:**
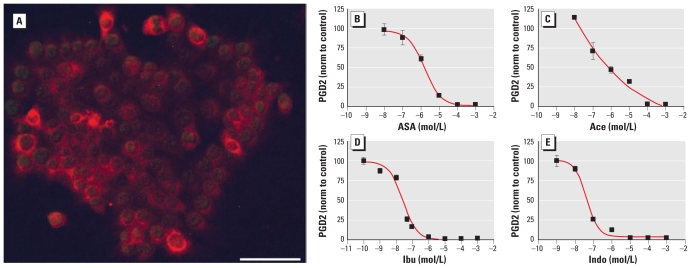
COX-2 enzyme expression and PGD2 secretion in the SC5 juvenile mouse Sertoli cell line. (*A*) Membrane-bound COX-2 enzyme located in the endoplasmic reticulum and nuclear envelope (weak 4′,6-diamidino-2-phenylindole (DAPI) nuclear counterstaining); bar = 50 μm. Inhibition of PGD2 secretion from mouse SC5 cells by ASA (*B*), Ace (*C*), Ibu (*D*), and Indo (*E*), normalized (norm) to control values. Data are mean ± SE for three experiments performed in triplicate.

**Figure 2:**
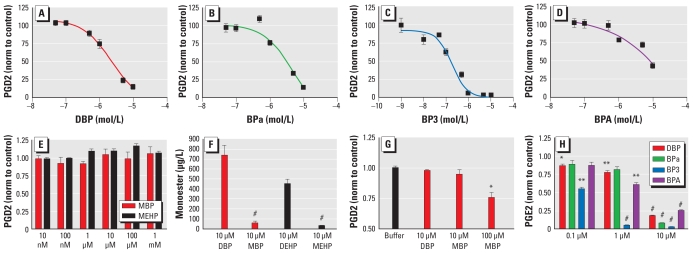
Endocrine disruptors inhibit PG synthesis in the SC5 juvenile mouse Sertoli cell line. DBP (*A*), BPa (*B*), BP3 (*C*), and BPA (*D*) dose-dependently inhibit secretion of PGD2 after incubation for 24 hr [normalized (norm) to control values]. (*E*) Incubation for 24 hr with MBP and MEHP, monoesters of DBP and DEHP, showed that they had no inhibitory effect on PGD2 secretion from SC5 cells. (*F*) LC-MS/MS analysis for MBP and MEHP after exposure to parental compounds DBP and DEHP and the monoesters revealed that MBP and MEHP were not taken up by SC5 cells. (*G*) Electroporation with MBP showed that the compound has inhibitory effect on PGD2 secretion from SC5 cells. (*H*) PGE2 is dose-dependently inhibited by DBP, BPa, BP3, and BPA in SC5 cells after 24 hr incubation. Data are mean ± SE for three experiments performed in triplicate. *p < 0.05, **p < 0.01, and ^#^*p* < 0.001, compared with controls by two-tailed Student’s *t*-test.

Overnight incubation of 10^5^ SC5 cells (Figure 1A) in a 12-well plate with 1 mL medium resulted in approximately 300 pg/mL PGD2 and approximately 15 ng/mL PGE2. This secretion was dose-dependently inhibited by Ace, ASA, ibuprofen (Ibu), and indomethacin (Indo) after 24 hr incubation (Figure 1B–E). Similar dose-dependent inhibition of PGD2 secretion from Sertoli cells was evident after incubation with many EDCs, including bisphenol A (BPA), genistein, diethylstilbestrol (DES), and flutamide (Figure 2A–D; for an extended list, see Table 1). We found no signs of cytotoxicity. The most potent inhibition of PGs occurred with benzophenone 3 (BP3), diisobutyl phthalate (DiBP), and isobutylparaben (iBPa), which were more potent than ASA and Ace. We saw no reduction in secretion of PGs after 24 hr incubation with natural estrogen and testosterones. Instead, testosterone, dihydrotestosterone, and tamoxifen, all at 10 μM, actually increased PG production.

### Effect of phthalate monoesters

MBP, the monoester of DBP, had no inhibitory effect on PGD2, nor did MEHP (Figure 2E) or DEHP (data not shown). This was puzzling because MBP, DEHP, and MEHP are all known to have endocrine-disrupting effects on male development (Scott et al. 2009). LC-MS/MS revealed that DBP and DEHP entered the cells, where they were converted to monoesters, whereas the monoesters MBP and MEHP were excluded probably because of their negative charge (Figure 2F). Because MBP has been argued to be the active metabolite of DBP, we electroporated 10 μM and 100 μM MBP into the SC5 cells, which at 100 μM resulted in a significant inhibition of PGD2 after 6 hr (Figure 2G). Thus, these results suggest that DBP passes into the cells, where it is metabolized to MBP, which (possibly together with DBP) subsequently inhibits PG synthesis. LC-MS/MS showed no other metabolites of DEHP in the cells except MEHP, indicating that the cells had a very limited capacity to create secondary metabolites such as 5-OH-MEHP, 5-OXO-MEHP, and 5-CX-MEPP (Koch et al. 2005).

**Figure 3:**
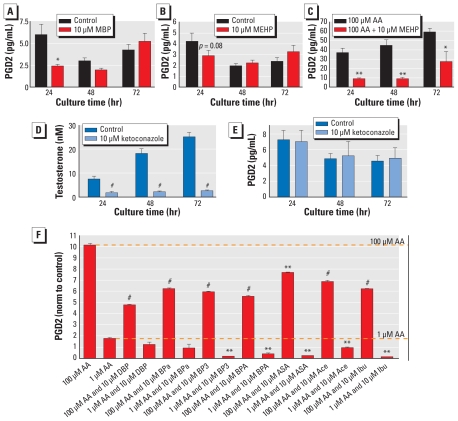
Inhibition of PG synthesis in fetal testes from GD14.5 rats (A–E) and SC5 juvenile mouse Sertoli cells (*F*). (*A*) MBP (10 μM) inhibits secretion of PGD2 after 24 hr culture. (*B*) MEHP (10 μM) weakly inhibits PGD2 secretion after 24 hr. (*C*) The inhibitory action of MEHP is evident after stimulation of PGD2 synthesis with 100 μM AA for all time points, also implying that the inhibition is downstream from AA. The CYP17 inhibitor ketoconazole reduced testosterone production (*D*) but did not affect PGD2 synthesis (*E*). (*F*) Stimulation of PGD2 secretion from SC5 cells with 1 and 100 μM AA is inhibited by DBP, BPa, BP3, and BPA, normalized (norm) to control values. Similar action is seen with pharmaceutical inhibitors ASA, Ace, and Ibu, indicating that DBP, BPa, BP3, and BPA are inhibiting the COX enzymes. Data are mean ± SE for three experiments performed in triplicate. *p < 0.05, **p < 0.01, and ^#^*p* < 0.001, compared with controls by two-tailed Student’s *t*-test.

To investigate whether MEHP and MBP could modulate PGD2 production in fetal testes, we incubated GD14.5 rat testes with 10 μM of either compound. PGD2 secretion was reduced after 24 hr for both compounds; however, the change was not statistically significant for MEHP (Figure 3A,B). After PGD2 production was increased by stimulation of the GD14.5 testes with 100 μM AA, 10 μM MEHP exposure resulted in significant inhibition of PGD2 secretion throughout 72 hr of culture (Figure 3C). The CYP17 inhibitor ketoconazole (Scott et al. 2009) reduced testosterone production without affecting PGD2 synthesis (Figure 3D,E).

### COX enzymes are the likely point of inhibition

We used the SC5 cell assay to investigate whether PGE2 synthesis also was inhibited by DBP, *n*-butylparaben (BPa), BP3, and BPA (Figure 2H). For all, we found dose responses similar to those for PGD2, implying that the point of inhibition is upstream from PGD2 and PGE2 synthases in the PG pathway. The previous experiments with GD14.5 rat testes showed that AA did not prevent MEHP-mediated inhibition of PGD2. To verify these results, we incubated SC5 cells for 24 hr with either Ace, ASA, Ibu, DBP, BPa, BP3, or BPA and then stimulated with 1 μM or 100 μM AA in medium containing the same compounds for 1 hr. The results showed that all compounds had an inhibitory effect (Figure 3F), signifying that the point of inhibition most likely is COX-1 and COX-2.

### The inhibitory effect of the EDCs is not mediated through the canonical PGD2 and PGE2 receptors

With data suggesting that the point of inhibition by the EDCs is the COX enzymes, we focused on the mode of action. Because DBP has some structural resemblance to PGs (Tavares et al. 1984), a mechanism through the PG receptors seemed plausible. However, exposure to BW245c, an agonist for PGD2 receptor (DP1) did not affect the synthesis of PGD2, and AH6809, an inhibitor of both DP1 and PGE2 receptor (EP1), had no effect on the inhibitory effect of DBP on PGD2 secretion from SC5 cells [see Supplemental Material, Figure 1a,b (doi:10.1289/ehp.1002635)], suggesting that the PGD2 and PGE2 receptors were not involved in the inhibitory action of the compounds.

### The inhibitory effect of the EDCs is not mediated through PPARs

Because phthalates, BPA, and other known EDCs are activators of PPARs (Diamanti-Kandarakis et al. 2009), we investigated whether the inhibitory effect was mediated through PPARs. Two different PPARδ (also known as PPARβ) agonists (GW0742 and GW501516) and a PPARδ antagonist (GSK0660) dose-dependently inhibited PGD2 secretion from SC5 cells [see Supplemental Material, Figure 1c–e (doi:10.1289/ehp.1002635)]. Moreover, the PPARγ and RXR agonists rosiglitazone and LGD1069 (see Supplemental Material, Figure 1f and Figure 1i, respectively) had similar but weaker effects, whereas two different PPARα agonists (613333 and GW590735) had no effect (see Supplemental Material, Figure 1g,h). Surprisingly, retroviral overexpression of *PPAR*δ, *PPAR*γ, and *PPAR*α in SC5 cells (see Supplemental Material, Figure 2a) resulted in no net change in PGD2 inhibition after incubation with DBP, BPa, BP3, or BPA (see Supplemental Material, Figure 2b). Lentivirus-mediated short hairpin RNA (shRNA) knockdown of *PPAR*δ or *PPAR*γ, which effectively reduced the respective mRNA levels (see Supplemental Material, Figure 2c), further indicated no association between the inhibitory effect of DBP, BPa, BP3, or BPA on PG synthesis and the *PPAR* genes (see Supplemental Material, Figure 2d).

As an independent confirmation of these data, we transfected SC5 cells with a PPAR-responsive luciferase reporter plasmid (TK-PPRE-luc), and the next day we exposed the cells to DBP, BP3, BPa, or *n*-propylparaben (PPa) and PPAR agonists or antagonists for 24 hr. The results showed no PPAR-activated transcription after exposure to DBP and BP3, whereas BPa and PPa slightly increased PPAR activity [see Supplemental Material, Figure 2e (doi:10.1289/ehp.1002635)]. Focusing on the mouse ligand-binding domain (LBD) of PPARδ and PPARγ, we transfected cells with PPARδ-LBD/Gal4 and PPARγ-LBD/Gal4 expression vectors together with a Gal4-responsive luciferase reporter plasmid; the next day cells were exposed to DBP, DiBP, BP3, PPa, or BPa. Again, we observed no increase in transcriptional activation (see Supplemental Material, Figure 2f,g), confirming that the inhibitory activity of the EDCs on PG synthesis is unlikely to be mediated by PPARs.

### The inhibitory effect of EDCs is not mediated by consistent changes in COX gene expression

Many of the compounds identified as PG synthesis inhibitors (Table 1) are also known to have estrogenic effects (Diamanti-Kandarakis et al. 2009). However, the lack of consistency between the strength of PG inhibition and the known estrogenic potency and lack of inhibition by 17β-estradiol and antiestrogens (i.e., ICI 182780, tamoxifen, and 4-hydroxy-tamoxifen) imply that the effect is not mediated through estrogen receptors. However, to further investigate the possible role of expression levels of *Cox1* and *Cox2* (*Pghs1* and *Pghs2*) genes in SC5 cells, we performed real-time PCR on RNA (complementary DNA) from cells exposed to some of the compounds that changed PG secretion. We observed no significant changes in expression levels for the two *Cox* genes, except for an increase in expression level after exposure to BP3 [see Supplemental Material, Table 2 (doi:10.1289/ehp.1002635)]. Thus, the inhibition of PG synthesis was not associated with decreased expression of the *COX* genes.

### EDCs interfere with PG secretion in human immune mast cells

**Figure 4:**
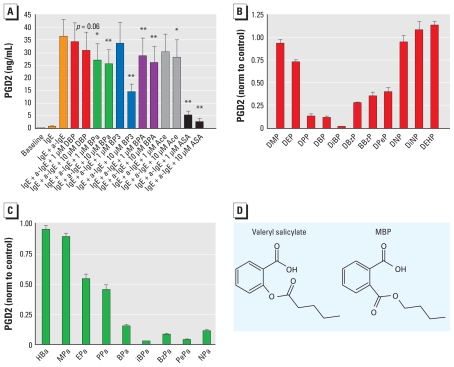
Endocrine disruptors share structural features with mild analgesics and inhibit PGD2 secretion from primary human mast cells. (*A*) Secretion of PGD2 from primary human mast cells after stimulation with IgE and anti-IgE is dose-dependently inhibited by exposure to DBP, BPa, BP3, BPA, ASA, and Ace (*n* = 8). (*B* and *C*) Secretion of PGD2 after exposure to different compounds (at 10 μM), indicating that compounds with an isobutyl side chain (DiBP and iBPa) cause the most potent inhibition (*n* = 3). Data are normalized (norm) to control values. (*D*) Phthalates share structural similarities with salicylates, here exemplified with valeryl salicylate and MBP. Data are mean ± SE. Abbreviations: BzPa, benzylparaben; DBzP, dibenzyl phthalate; DEHP, diethylhexyl phthalate; DEP, diethyl phthalate; DiBP, diisobutyl phthalate; DiNP, di-isononyl phthalate; DMP, dimethyl phthalate; DNP, di-*n*-nonyl phthalate; DPeP, di-*n*-pentyl phthalate; DPP, di-*n*-propyl phthalate; EPa, ethylparaben; HBa, 4-hydroxy benzoic acid; iBPa, isobutylparaben, MPa, methylparaben, NPa, *n*-nonylparaben; PePa, *n*-pentylparaben; PPa, *n*-propylparaben. *p < 0.05, **p < 0.01, compared with controls by two-tailed Student’s *t*-test.

To test whether human PG synthesis also was inhibited, we focused on the immune system, where PGD2 secretion from mast cells plays a key role in immediate-type hypersensitivity reactions such as anaphylactic reaction, acute asthma, and allergic rhinitis (Ishizaka et al. 1983). Primary *in vitro* differentiated human mast cells (10^5^) were sensitized with human myeloma IgE and exposed to test compounds for 24 hr, followed by activation of the surface receptor FcɛRI with anti-IgE. After 30 min the cells had secreted approximately 25 ng/mL PGD2 on average. This PGD2 pulse was dose-dependently inhibited by Ace, ASA, DBP, BPa, BP3, and BPA (Figure 4A).

### Modeling suggests that the EDCs directly inhibit COX enzymes

Phthalates and parabens are structurally similar compounds, and there seemed to be a correlation between the length of the alkyl side chain of DiBP and iBPa and the potency of inhibition (Figure 4B,C and Table 1). For example, for both phthalates and parabens, compounds with isobutyl side chains had the most pronounced inhibitory effects, which suggests a similar mode of action. Because of the high level of structural similarity between phthalates and some commercial COX inhibitors, as exemplified by MBP and valeryl salicylate (Figure 4D), we conducted simulation of the compounds into the binding pocket of COX-2. The binding site of COX enzymes is a hydrophobic channel with possible hydrogen bonding at the mouth with Tyr355 and Arg120 and at the bottom of the channel with Tyr385. The ASA acetylation site, Ser530, is positioned below Tyr385 and is another possible target for hydrogen bonding (Kurumbail et al. 1996; Luong et al. 1996; Picot et al. 1994). Modeling showed that mean predicted dissociation constant (predicted p*K*_i_) scores of the binding of DBP, BPa, BP3, and BPA simulated into the COX-2 active site were higher than the Andrews mean p*K*_i_ [see Supplemental Material, Table 3a (doi:10.1289/ehp.1002635)] that estimates docking in a random binding site, implying that these compounds could be accommodated in the ligand binding pocket of COX-2.

The simulations also provided an explanation for the observed differences in potencies seen for the phthalates and parabens. Placing molecules of each paraben from methyl to butyl side chains (*n* = 1–4) within the active site of COX-2, in a position allowing the best match with hydrogen bonding to Ser530 and Tyr385 by the carbonyl groups in the ester bonds, showed that differences in the predicted p*K*_i_ scores [see Supplemental Material, Table 3a (doi:10.1289/ehp.002635)] were similar to the observed differences in half-maximal inhibitory concentration (IC_50_) (Table 1). We obtained similar results with predictions for both phthalate diesters and monoesters. Thus, the binding affinity of the phthalates and parabens can be explained by hydrophobic and van der Waals interactions in the channel lined with hydrophobic residues. The strength of the interactions increases with increasing length of the alkyl group and with branching, as demonstrated by the high hydrophobic interaction potential of compounds with an isobutyl side chain (DiBP and iBPa). The importance of the hydrophobic binding for ligand–COX-2 interaction is well documented (Soliva et al. 2003). The simulation also suggests that with increasing side chain length (*n* > 5), compounds begin to get too large for the binding site, which reduces the affinity, possibly explaining the decrease in potency of longer chained phthalates such as DEHP, di-*n*-nonyl phthalate, and diisononyl phthalate. Consistent with this, the modeling also suggests that for the larger phthalates, metabolites are more likely candidates for inhibitory action than are the parent compounds. Hence, the secondary metabolites of DEHP (5-OH-MEHP, 5-OXO-MEHP, and 5-CX-MEPP) all had higher affinity in the model than did DEHP and MEHP, because they may form hydrogen bonds both with Arg120 and Tyr355 at the mouth of the channel and with Tyr385 at the bottom [see Supplemental Material, Table 3b), supporting the notion that the active metabolite of DEHP is not MEHP but one of the secondary compounds that were not detected in SC5 cells.

## Discussion

In this study we found that many putative EDCs inhibit the PG pathway. Using various experiments, including viral transduction and transfection assays, we observed that PG inhibition is independent of PGD and PGE receptors (DP and EP) and PPAR receptors and that it occurs without changes in the expression of the *Cox* genes. Instead, our data suggest that the compounds directly interfere with the activity of the COX enzymes in a manner similar to mild analgesics such as ASA, Ace, and Ibu.

Sertoli cells have been hypothesized to be a central point of endocrine disruption during prenatal development of the testes (Skakkebaek et al. 2001), and signaling from fetal Sertoli cells is sensitive to PGs. PGD2 has been shown to be involved in early sexual differentiation (Adams and McLaren 2002), and other studies have shown that the PG pathway in general is important for the masculinization of the male reproductive tract (Gupta 1989; Gupta and Bentlejewski 1992; Gupta and Goldman 1986). We used the SC5 juvenile mouse Sertoli cell line to screen for inhibition of PG synthesis because it produces high amounts of PGs without prior stimulation.

The monoesters MBP and MEHP did not enter the SC5 cells as readily as did the diesters (DBP and DEHP), possibly because the charged molecules cannot pass the plasma membrane. Accordingly, we found no effect on PGD2 secretion from the cells. However, electroporation of MBP into the cells showed that MBP does inhibit PGD2 secretion. We cannot explain why MBP inhibited the secretion of PGD2 in the fetal rat testes, but we speculate that MBP uptake may differ from that of SC5 cells.

Neither DEHP nor its primary metabolite, MEHP, inhibited the secretion PGD2 from SC5 cells. LC-MS/MS showed that the secondary metabolites were not detectable in the cells, and modeling suggested that DEHP and MEHP did not fit well into the active site of the COX-2 enzymes. However, exposing GD14.5 fetal rat testes to MEHP reduced PGD2 secretion, which became significant after stimulation of PG synthesis with AA. We therefore speculate that the responsible metabolite is not MEHP, but 5-OH-MEHP, the first metabolite of MEHP, which has also been found to have an antiandrogenic effect in fetal testis (Chauvigne et al. 2009) but is not detectable in SC5 cells.

All the investigated compounds that had an inhibitory action on PG synthesis have one or more apolar benzene rings, a structural feature known to play a central role in COX inhibition by pharmaceutical inhibitors (Soliva et al. 2003). This can be attributed to interactions between the apolar rings and the hydrophobic amino acids lining the channel of COX enzymes, an interaction predicted to stabilize the binding with van der Waals interactions. Supporting this concept, EDCs without aromatic rings, such as perfluorooctanoic acid, perfluorosulfonic acid, and citral, showed no PG inhibitory effect in SC5 cells (data not shown). Furthermore, phenol alone and substituted phenols in general inhibit COX enzyme activity through binding in the active site, thereby obstructing enzyme kinetics (Hsuanyu and Dunford 1992), which signifies the inhibitory effect of the benzene group on the PG pathway. The best known of these substituted phenols is Ace, but others are catechol, catecholamines (e.g., adrenalin), hydroquinone, eugenol (the principal component of natural analgesic clove oil), and resveratrol (an active component of red wine) (Graham et al. 2001; Zykova et al. 2008). Our data suggest that parabens and more complex molecules with multiple phenol groups, such as BP3, BPA, DES, and genistein, should be added to the already characterized substituted phenols with inhibitory effects on PG synthesis.

Tavares and Vine (1985) reported that certain phthalates could interfere with formation of products from the COX and lipoxygenase enzymes in rat peritoneal leucocytes. In the present study we focused exclusively on the PG pathway and therefore did not determine whether lipoxygenases are also inhibited. Fujimoto et al. (2005) measured PG inhibition in rabbit kidney medulla microsomes and concluded that nonylphenol directly inhibits COX activity. However, they also found that BPA and DBP did not have an inhibitory effect on the PG cascade, which is in conflict with our results and results from a study of peritoneal leukocytes (Tavares and Vine 1985). We cannot explain this discrepancy, but it may be related to the use of different experimental designs; also, the proposed binding of EDCs to the COX enzymes should be confirmed using alternative experimental methods.

Genistein and other isoflavones have been reported to decrease PG synthesis in a neck cancer cell line (Ye et al. 2004), in prostate cancer cells (Swami et al. 2007, 2009), and in prostate cancer patients (Swami et al. 2009), where the compounds reportedly blocked the development and progression of prostate cancer. In a recent study Swami et al. (2009) reported that genistein decreased expression of COX enzymes without affecting COX promoter activity. The authors argued that genistein most likely inhibits PG synthesis through repression of transcriptional activation by growth factors (Swami et al. 2009). However, Ye et al. (2004) reported that genistein inhibited the PG pathway without affecting *COX* gene expression, consistent with our data suggesting that inhibition results from direct effects on COX enzyme activity. Interestingly, genistein has previously been reported to have dual effects in rats (Eustache et al. 2009). Low doses of genistein (1 mg/kg/day) and vinclozolin (1 mg/kg/day) were more antiandrogenic when added simultaneously than when added one at the time, but at higher doses (10 mg/kg/day genistein and 30 mg/kg/day vinclozolin) the antiandrogenic effect of vinclozolin was attenuated by genistein (Eustache et al. 2009). Although speculative, it is possible that the low-dose effect could be due to an antiandrogenic effect by inhibition of PG synthesis.

Finally, if PG inhibition is involved in the mode of action of some EDCs, it raises the worrying possibility that pharmaceutical PG inhibitors such as ASA, Ace, and Ibu may act as endocrine disruptors. In our investigation of this we found that Ace indeed reduced the anogenital distance in rat pups after prenatal exposure and that prenatal exposure to ASA reduced testosterone production in fetal testis (Kristensen et al. 2010). Accordingly, in a prospective birth cohort study we found that use of ASA, Ace, and Ibu was associated with cryptorchidism in newborn boys (Kristensen et al. 2010), the best-known risk factor for reduced fertility and testicular germ cell cancers in adulthood (Boisen et al. 2004).

If inhibition of PG synthesis is the mechanism of antiandrogenicity of compounds such as phthalates, chronic inhibition of the PG pathway by a large number of EDCs combined with several short-term high-dose exposures to mild analgesics could have an impact on male reproductive health. Furthermore, a growing number of studies has shown that prenatal and early childhood exposure to Ace is associated with atopic diseases (Beasley et al. 2008; Perzanowski et al. 2010; Rebordosa et al. 2008; Shaheen et al. 2002, 2005). Data in the present study show that some of the EDCs are more potent inhibitors of human primary mast cell responses after activation than is Ace; thus, this may suggest a link between exposure to environmental pollutants and disturbances of the immune system.

To conclude, the present study shows an unrecognized point of endocrine disruption through inhibtion of PG synthesis. Therefore, more research is needed to investigate whether EDCs could play a role in the increase of immunological and reproductive diseases through inhibition of the PG pathway.

## Figures and Tables

**Table 1:** IC_50_ values for inhibition of PG secretion from mouse SC5 cells.

Test compound	Abbreviation	IC_50_
Pharmaceutical inhibitors		

Acetyl salicylate (aspirin)	ASA	1.64 × 10^−6^
Acetaminophen/paracetamol	Ace	3.82 × 10^−7^
Ibuprofen	Ibu	1.12 × 10^−8^
Indomethacin	Indo	4.24 × 10^−7^

Phthalates		

Dimethyl phthalate	DMP	No effect
Diethyl phthalate	DEP	1.9 × 10^−5^
Di-*n*-propyl phthalate	DPP	2.1 × 10^−6^
Di-*n*-butyl phthalate	DBP	2.11 × 10^−6^
Diisobutyl phthalate	DiBP	1.01 × 10^−6^
Butylbenzyl phthalate	BBzP	2.45 × 10^−5^
Di-*n*-pentyl phthalate	DPeP	1.49 × 10^−4^
Di-*n*-benzyl phthalate	DBzP	4.17 × 10^−4^
Di-*n*-nonyl phthalate	DNP	No effect
Diisononyl phthalate	DiNP	No effect
Di-2-ethylhexyl phthalate	DEHP	No effect

Parabens		

Ethylparaben	EPa	7.59 × 10^−6^
*n*-Propylparaben	PPa	2.85 × 10^−6^
*n*-Butylparaben	BPa	2.43 × 10^−6^
Isobutylparaben	iBPa	1.09 × 10^−6^
*n*-Pentylparaben	PePa	4.83 × 10^−7^
Benzylparaben	BzPa	1.30 × 10^−6^
*n*-Nonylparaben	NPa	2.91 × 10^−6^

Benzophenones		

Benzophenone 3	BP3	1.97 × 10^−7^
Benzophenone 7		1.20 × 10^−6^
Benzophenone 4		4.56 × 10^−5^
Benzophenone 12		6.94 × 10^−5^

Estrogen and estrogenic compounds		

17β-Estradiol		11.28 × 10^−2^
Diethylstilbestrol	DES	2.72 × 10^−5^
Zearalenol		3.18 × 10^−6^
Genistein		4.28 × 10^−4^
Bisphenol A	BPA	2.72 × 10^−6^
Coumestrol		1.25 × 10^−5^
Nonylphenol		No effect

Antiestrogenic compounds		

Tamoxifen		Increase at 10 μM
4-OH tamoxifen		Increase at 10 μM
ICI 182780	ICI	No effect

Androgens and antiandrogen		

Testosterone		Increase at 10 μM
Dihydrotestosterone		Increase at 10 μM
Flutamide		1.87 × 10^−6^

IC_50_, half-maximal inhibitory concentration.
